# Clinical and preclinical studies of mesenchymal stem cells to alleviate peritoneal fibrosis

**DOI:** 10.1186/s13287-024-03849-3

**Published:** 2024-07-30

**Authors:** Lingqian Zheng, Wenmin Chen, Kaijin Yao, Yina Xie, Chunling Liao, Tianbiao Zhou

**Affiliations:** Department of Nephrology, the Second Affiliated Hospital of Shantou University Medical College, No. 69 Dongsha Road, Shantou, 515041 China

**Keywords:** Mesenchymal stem cells, Peritoneal fibrosis, Peritoneal dialysis, Exosomes

## Abstract

Peritoneal dialysis is an important part of end-stage kidney disease replacement therapy. However, prolonged peritoneal dialysis can result in peritoneal fibrosis and ultrafiltration failure, forcing patients to withdraw from peritoneal dialysis treatment. Therefore, there is an urgent need for some effective measures to alleviate the occurrence and progression of peritoneal fibrosis. Mesenchymal stem cells play a crucial role in immunomodulation and antifibrosis. Numerous studies have investigated the fact that mesenchymal stem cells can ameliorate peritoneal fibrosis mainly through the paracrine pathway. It has been discovered that mesenchymal stem cells participate in the improvement of peritoneal fibrosis involving the following signaling pathways: TGF-β/Smad signaling pathway, AKT/FOXO signaling pathway, Wnt/β-catenin signaling pathway, TLR/NF-κB signaling pathway. Additionally, in vitro experiments, mesenchymal stem cells have been shown to decrease mesothelial cell death and promote proliferation. In animal models, mesenchymal stem cells can enhance peritoneal function by reducing inflammation, neovascularization, and peritoneal thickness. Mesenchymal stem cell therapy has been demonstrated in clinical trials to improve peritoneal function and reduce peritoneal fibrosis, thus improving the life quality of peritoneal dialysis patients.

## Introduction

**Fig. 1:**
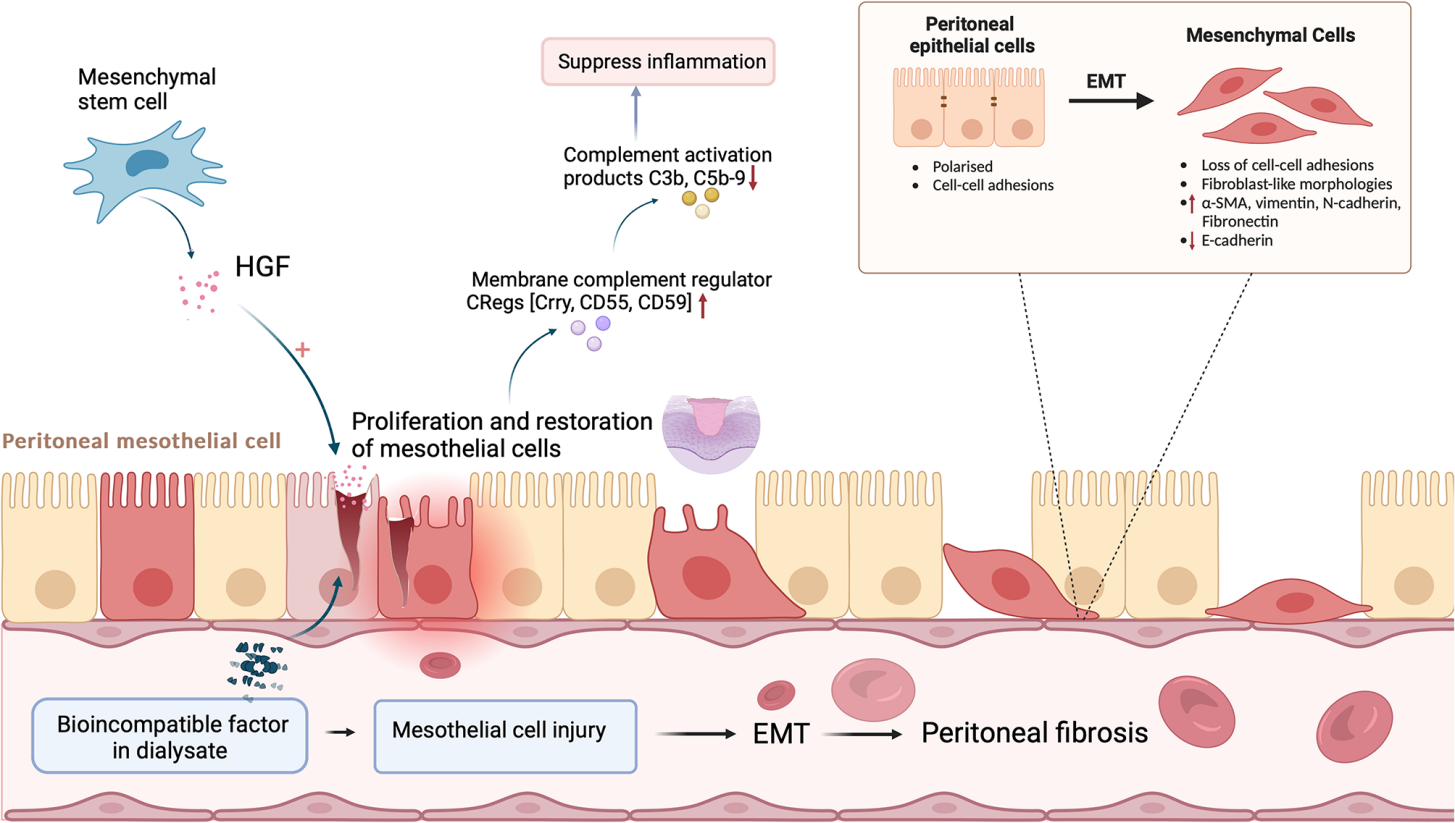
Epithelial-mesenchymal transition (EMT) of peritoneal mesothelial cells. Biocompatible factors in dialysate may contribute to mesothelial cell injury, leading to EMT. EMT is an important phase in the development of peritoneal fibrosis, involving the transformation of an epithelial phenotype into a mesenchymal phenotype. During this process, cells lose their polarity, adherence, and tight junctions, resulting in a fibroblast-like appearance and mesenchymal cell phenotypic expression (including upregulation of α-SMA, vimentin, N-cadherin, fibronectin, and downregulation of E-cadherin). Mesenchymal stem cells (MSCs) could secrete HGF (hepatocyte growth factor) to promote the proliferation and restoration of mesothelial cells, which could express a large number of membrane complement regulators (Crry, CD55), further decreasing the complement activation product C3b, C5b-9 to inhibit inflammation. Created by BioRender.com

Peritoneal dialysis (PD) is an essential renal replacement treatment for patients with end-stage renal disease (ESRD). Around the world, more than 196,000 individuals with ESRD are presently undergoing PD treatment [1]. In contrast to conventional hemodialysis, PD provides several benefits, including low medical resources and cost savings due to its straightforward operation, effective removal of mesomolecular molecules, minor loss of remaining renal functions, and steady hemodynamics [2]. However, PD patients are persistently exposed to biologically incompatible dialysis solutions with high concentrations of glucose, which may contribute to losing normal morphology and function in peritoneal mesothelial cells, resulting in ultrafiltration failure (UFF) and peritoneal fibrosis (PF) [3]. PF is a severe consequence of the PD procedure. It is characterized by the loss of peritoneal mesothelial cells, the aberrant proliferation of α-SMA-positive myofibroblasts, a noticeable buildup of collagen, and a gradual increase in the thickness of the submesothelial compact zone [4, 5]. Peritoneal function can be improved by controlling the occurrence of PF [6–8]. Epithelial-mesenchymal transition (EMT) was an initiating and reversible step of PF [9]. EMT is a cellular transdifferentiation process that involves the transformation of an epithelial phenotype into a mesenchymal phenotype. During this process, the cells lose their polarity, adherence, and tight junctions, resulting in a fibroblast-like appearance and mesenchymal cell phenotype, leading to PF and UF [2, 10]. Therefore, exploring the mechanism of EMT is crucial for alleviating PF in PD patients (Fig. 1).

Mesenchymal stem cells (MSCs) are multipotent adult stem cells that can be isolated from multiple kinds of tissues, including bone marrow, adipose tissue, umbilical cord blood, and the placenta [11–13]. MSCs have regenerative, immunomodulatory, and antifibrotic capabilities [14]. As for antifibrotic ability, various studies have investigated the fact that MSCs can ameliorate fibrosis in the liver, heart, lung, and kidney among different animal models [15–18]. Additionally, MSCs can repair tissue damage, but their mechanism is controversial. Initially, it was thought that MSCs repair tissues by engrafting and differentiating to replace injured cells [19–23]. Nowadays, many studies have proved that MSCs exert their beneficial effects by secreting cytokines and growth factors through the paracrine pathway or cell-to-cell contacts. The cytokines, such as TNF-α-stimulated gene 6 protein (TSG-6) [6, 24], VEGF_ENREF_38 [25], and PDGF [26], can alter the tissue microenvironment to repair tissue injury. It is worth noting that the cytokines released by MSCs have the potential to be amplified through crosstalk with injured cells, resulting in the expression of even more therapeutic factors [23, 27]. Exosomes are tiny vesicles secreted by MSCs via the paracrine pathway that contain a variety of biomolecules, such as proteins, non-coding RNA, DNA, and mRNA, and act as important carriers in cell communication [28]. Recent research has shown that exosomes derived from hUMSCs (human umbilical cord-derived mesenchymal stem cells) can reduce the fibrosis of organs, such as the liver, lung, and kidney [29–31].

This review summarises the latest studies on MSCs in the in three parts: (1) the mechanism of MSCs in treating PF, (2) in vitro experiments, (3) animal experiments, and (4) clinical trials. In addition, we discuss the different sources of MSC, which may lead to different results. Different injection methods may have different effects on peritoneal adhesion, possible problems in applying results of animal experiments to clinical studies, and the current research progress in combining biotechniques with stem cell therapy. This review will help advance the development of regenerative medicine and provide a new direction for cell therapy for human disease. The basic characteristics of the included literature are presented in Tables 1, 2 and 3.

**Table 1:** Summary of in vitro experiments on the effects of mesenchymal stem cells on peritoneal mesothelial cells

Author, year	Cell model	Stem cell type	group	Handling methods	Treatment effect	Outcome
Wang et al. (2012) [72]	Mechanical scraping RPMCs model	Rat-BM-MSCs	1. Control 2. MSCs co-cultured	Co-cultured RPMCs with MSCs using a Transwell system.	↑: Migration, Proliferation	MSCs promote the repair and proliferation of injured RPMCs in vitro.
Ueno et al. (2013) [60]	Glucose-induced HPMCs	Human-BM-MSCs	1. Control 2. Glucose + vehicle 3. Glucose + MSCs	Co-culture Human MSCs with HPMCs using a Transwell system.	Glucose + MSCs treated: ↓: TGF-β1, Fibronectin	MSCs can secrete HGF through the paracrine pathway and improve TGF-β1-stimulated EMT in HPMCs through the TGF-β1/Smad signaling pathway.
	TGF-β1-stimulated EMT of HPMCs	MSC-CM	1. Control 2. MSC-CM 3. TGF-β1 4. TGF-β1 + MSC-CM	Co-culture HPMCs with MSC-CM and then treated with TGF-β1.	↓: α-SMA, Smad2, Fibroblastic change of HPMCs, decrease in ZO-1	
	TGF-β1-stimulated EMT of HPMCs	MSC-CM	1. MSC-CM 2. HPMC-CM	Culture HPMCs and MSCs in 0.1% FBS containing Dulbecco’smodified Eagle’s medium, measure the concentrations of HGF in MSC-CM and HPMC-CM.	MSC-CM treated: ↑: HGF HPMC-CM treated: (below the detection level)	
	TGF-β1-stimulate EMT of HPMCs	1. MSC-CM 2. MSC-CM + HGF- Ab	1. TGF-β1 2. TGF-β1 + HGF-Ab 3. TGF-β1 + MSC-CM 4. TGF-β1 + MSC-CM + HGF-Ab	MSC-CM was treated with or without HGF-antibody and then cultured with TGF-β1-stimulate HPMCs.	1. TGF-β1 + MSC-CM-treated: ↓: pSmad2 2. TGF-β1 + MSC-CM + HGF-Ab treated: ↑: pSmad2	
KIM et al. (2014) [64]	Mechanical scraping RPMCs	Rat ASCs	1. Rat ASCs (+) 2. Rat ASCs (-)	Co-culture RPMCs with Rat ASCs.	↑: the number of mesothelial cells	Rat ASCs may secrete HGF by paracrine pathway to promote the proliferation and restoration of injured RPMCs.
	Mechanical scraping RPMCs	Rat-ASCs-CM	1. Rat-ASCs-CM 2. Rat-MC-CM	Co-culture RPMCs with Rat-ASCs-CM and Rat- MC-CM.	↑: the restoration of injured RPMCs monolayers	
	RPMCs	Recombinant rat HGF		RPMCs are stimulated in the medium with different amounts of recombinant rat HGF.	↑: Proliferation of RPMCs	
Li et al. (2018) [75]	TGF-β1-induced RPMCs	hUMSCs; hUMSCs-CM	1. Normal RPMSs 2. RPMCs + TGF-β1 3. RPMCs + TGF-β1 + hUMSCs 4. Normal hUMSCs 5. hUMSCs + TGF-β1	TGF-β1-induced RPMCs incubate with hUMSCs or hUMSCs-CM.	hUMSCs or hUMSCs-CM treated: ↑: miR-153-3p, E-cadherin ↓: Snail 1, α-SMA	hUMSCs may inhibit EMT through upregulate miR-153p.
Zhou et al. (2019) [71]	H2O2-induced apoptosis in HPMCs	pMSC-CM	1. Control 2. H_2_O_2_ 3. H_2_O_2_ + 25% pMSC-CM 4. H_2_O_2_ + 50% pMSC-CM	Incubation H_2_O_2_-induced apoptosis in HPMCs with pMSC-CM.	pMSC-CM treated: ↓: cell apoptosis, proinflammatory- factors (Such as CXCL6, NOS2, IL1RN, CCL5, NR3C1) ↑: anti-inflammatory factors (CCR1, CCR4, IL9, IL-10)	pMSC-CM can prevent cell death in HPMCs and down-regulate proinflammatory while upregulating anti-inflammatory gene expression in activated THP1 cells.
	PDS-induced apoptosis in HPMCs	pMSC-CM	1. Control 2. PDS 3. PDS + 50% pMSC-CM	Incubation PDS-induced apoptosis in HPMCs with pMSC-CM.		
	PMA/LPS-stimulated THP1 cells	pMSC-CM	PCR array analysis	Incubation PMA/LPS-activated THP1 cells in pMSC-CM by PCR array analysis.		
Guo et al. (2020) [73]	TGF-β1-induced Met-5 A cells	hUMSCs; SIRT1-hUMSCs	1. Control 2. TGF-β1 3. TGF-β1 + hUMSCs 4. TGF-β1 + SIRT1-hUMSCs	Co-culture hUMSCs and SIRT1-hUMSCs with TGF-β1-induced Met-5 A cells.	hUMSCs and SIRT1-hUMSCs treated: ↓: Fibronectin, α-SMA, and Snail ↑: E-cadherin	SIRT1-hUMSCs markedly inhibit TGF-β1 induced EMT of Met-5 A cells compare to hUMSCs.
Yang et al. (2021) [7]	LPS induced NR8383 macrophages	ADSC-CM; BM-MSC-CM	1. LPS 2. IMDM 3. BM-MSC-CM 4. ADSC-CM	NR8383 macrophages were treated with LPS and cultured with IMDM, ADSC-CM, or BM-MSC-CM.	ADSC-CM and BM-MSC-CM treated: →: iNOS ↑: Arg-1	ADSCs secreted higher levels of IL-6 and were more able to upregulate the level of Arg-1 in macrophages than BM-MSCs.
	TGF-β1 stimulated ADSCs and BM-MSCs	ADSCs; BM-MSCs	1. ADSC-CM 2. BM-MSC-CM	ADSCs and BM-MSCs was stimulated with the TGF-β1 and then collect the supernatant.	↑: IL-6	
Nagasaki et al. (2021) [41]	TGF-β1 stimulated HPMCs	SF-MSCs; 10%MSCs	1. Control 2. SF-MSCs 3. 10%MSCs	HPMCs treated with TGF-β1 cocultured with SF-MSCs, 10% MSCs, and control medium.	SF-MSCs or 10%MSCs treated: ↓: pSmad2, pSmad3, α-SMA ↑: Proliferative activity of MSCs	SF-MSCs and 10% MSC can both suppress the TGF-β1/Smad pathway with no difference. Serum-free medium can greatly improve the proliferative activity of MSCs.
Du et al. (2021) [70]	PDS-induced HPMCs death	pMSCs-CM; hUMSCs-CM	1. Control 2. PDS 3. PDS + pMSCs 4. PDS + hUMSCs	Co-culture HPMCs with pMSCs-CM or hUMSCs-CM, measure the survival of HPMCs exposed to PDS.	1. pMSCs-CM treated: ↓: cell apoptosis ↑: cell viability 2. UC-MSCs-CM treated: →: cell apoptosis →: cell viability	The pMSCs-CM can significantly inhibit cell apoptosis and increase the viability compared to hUMSCs-CM. The differentiation of osteocytes, adipocytes and chondrocytes are similar. The secretions produced by pMSCs and hUMSCs exhibit an equal ability to inhibit NOS-2 activity in inactivated THP1 cells.
	PMA/LPS-activated THP1 cells	pMSCs-CM hUMSCs-CM	1. Control 2. Medium + PMA/LPS 3. Medium + PMA/LPS + pMSCs 4. Medium + PMA/LPS +hUMSCs	pMSCs-CM or hUMSCs-CM were applied to incubate the activated THP-1 cells.	pMSCs-CM or hUMSCs-CM treated: ↓: upregulation of NOS2 ↓: NO production	
Huang et al. (2023) [68]	HG-induced HPMCs	hUMSCs-CM; hUMSC-Exo	1. Control 2. HG 3. HG + CM 4. HG + Exo	Coculture HG-induced HPMCs with hUMSCs-CM and hUMSC-Exo.	hUMSCs-CM or hUMSC-Exo treated: ↓: α-SMA, vimentin, miR-21, Wnt3a, β-catenin ↑: E-cadherin, lncRNA-GAS5, PTEN proteins	Exosomal lncRNA GAS5 from hUMSCs reduce EMT of HPMCs via the Wnt/β-catenin pathway by down-regulating miR21 and up-regulating PTEN.
	HG-induced HPMCs	GAS5i-exo	1. Control 2. HG 3. HG + NC GAS5i-exo 4. HG + GAS5i-exo	To confirm that exo-lncRNA GAS5 from the hUMSCs regulates HPMCs’ EMT, use GAS5 iRNA transfect with hUMSCs.	GAS5i-exo treated: ↓: E-cadherin, lncRNA GAS5, PTEN ↑: α-SMA, vimentin, miR-21, Wnt3a, β-catenin	
Jiao et al. (2023) [67]	HG-stimulated HMrSV5	hUMSC-CM; hUMSC-Exos	1. Control 2. HG 3. HG + CM 4. HG + Exo	HG stimulated HMrSV5 treated with hUMSC-CM and hUMSC-Exo.	hUMSC-CM or hUMSC-Exos treated: ↓: miR3149, α-SMA, Vimentin ↑: Inc-CDHR, E-cadherin	Exosomal lnc-CDHR of hUMSCs competitively bind to miR-3149 and regulate PTEN to inhibit HG-induced EMT. Exo-lnc-CDHR could down-regulate miR-3149 and upregulate PTEN via the AKT/FOXO pathway to reduce peritoneal fibrosis.
	HG-stimulated HMrSV5	hUMSC-CDHR siRNA exos	1. Control 2. HG 3. HG + NC exo 4. HG + CDHR-siRNAexo	Use Inc-CDHR siRNA to transfect hUMSCs.	hUMSC-CDHR siRNA exos treated: ↓: Inc-CDHR, E-cadherin, FOXO3a ↑: miR3149, α-SMA, Vimentin	
	HG-stimulated HMrSV5	pc-DNA CDHR	1. Control 2. HG 3. HG + NC CDHR 4. HG + CDHR	Use Pc-DNA CDHR transfect HG-stimulated HMrSV5.	HG + pc-DNA-CDHR treated: ↓: miR3149, ɑ-SMA, Vimentin ↑: E-cadherin, Inc-CDHR, FOXO3a	
	HG-stimulated HMrSV5	miR3149 inhibitor	1. Control 2. HG 3. HG + NC miR3149 4. HG + miR3149 inhibitor	Transfect the miR-3149 inhibitor into HG-stimulate HMrSV5.	HG + miR3149 inhibitor treated: ↓: miR3149, ɑ-SMA, vimentin, pAKT/AKT ↑: E-cadherin, FOXO3a, Inc-CDHR	
	HG-stimulated HMrSV5	Inc-CDHR and miR3149 mimic	1. Control 2. HG 3. HG + CDHR 4. HG + CDHR + miR3149mimic	Simultaneous transfect Inc-CDHR and miR3149 mimic into HG-stimulate HMrSV5.	HG + CDHR + miR3149mimic treated: ↓: E-cadherin, Inc-CDHR, PTEN ↑: miR3149, ɑ-SMA, vimentin, pAKT/AKT	

Abbreviation; ↓: down-regulation; ↑: improve the indicators; →: no significant change; RPMCs: rat peritoneal mesothelial cells; BM-MSCs: bone marrow mesenchymal stem cells; MSCs: mesenchymal stem cells; TGF-β1: transforming growth factor-β1; HPMCs: human peritoneal mesothelial cells; EMT: epithelial-to-mesenchymal transition; CM: conditional medium; ZO-1: zonula occludens-1; HGF: hepatocyte growth factor; BMP-7: bone morphogenic protein 7; Rat-MC-CM: rat mesothelial-conditional medium; Met-5 A: an immortalized human pleural mesothelial line; LPS: lipopolysaccharides; Arg-1:Arginine-1; iNOS: inducible nitric oxide synthase; IL-6: interleukin 6; SF-MSCs: MSCs in serum-free medium; 10%MSCs: MSCs cultured in medium containing 10% fetal bovine serum; PDS: PD solution; hUMSC: human umbilical cord-derived MSCs; THP-1 cells: a human monocytic cell line; HG: High concentration of glucose; Exo: exosomes; HMrSV5: Human peritoneal mesothelial cell line; NC: negative control

**Table 3:** Summary of clinical trials of mesenchymal stem cells for the treatment of peritoneal fibrosis

Author, year	Patients	Research type	Stem cell type	group	Handling methods	Treatment effect
Alatab et al. (2017) [85]	CAPD more than two years and have UFF (*n * = 9)	A prospective study, open-label, non-randomized, phase I trial	AD-MSCs	Self-control study	1.2 ± 0.1 × 10^6^ cell/kg of AD-MSCs via cubital vein	↓: BMI, D/Pcr ↑: UF
Jiang et al. (2017) [83]	ESRD Patients with CAPD treatment (*n* = 24)	Self-control study	hUMSCs	Group1: before using hUMSCs; Group2: at three months after using hUMSCs; Gruop3: at four years after using hUMSCs	2 × 10^7^ hUMSCs via peripheral vein and renal artery intervention	↑: Hb, serum EPO, serum Alb, urine volume, cystatin C ↓: Hs-CRP
Ahmadi et al. (2023) [8]	CAPD more than two years and have UFF (*n * = 9)	A prospective, open-label, pilot study	AD-MSCs	Self-control study	Patients received 1.2 ± 0.1 × 10^6^ cell/kg of AD-MSCs via cubital vein	↓: CA125, TGF-β, α-SMA, FSP-1 ↑: FWT, UFSP, UFT, OCG, D/Pcr, Dt/D0 glucose

Abbreviation; ↓: down-regulation; ↑: up-regulation; AD-MSCs: adipose-derived mesenchymal stem cells; BMI: body mass index; D/P cr: dialysate to plasma ratio of creatinin; UF: ultrafiltration; Hb: hemoglobin; EPO: erythropoietin; Alb: albumin; Hs-CRP: high-sensitivity C-reactive protein; CA125: mesothelial marker of cancer antigen 125; TGF-β: transforming growth factor-β; α-SMA: α-Smooth muscle actin; FSP-1: fibroblast-specific protein-1; FWT: free water transport; UFSP: ultrafiltration-small pore; UFT: ultrafiltration total; OCG: osmotic conductance to glucose; Dt/D0: dialysate glucose concentration at the end of the test / “fresh” solution glucose concentration; hUMSCs: human umbilical cord blood mesenchymal stem cells; CAPD: continuous ambulatory peritoneal dialysis

## Mechanism

### Promote macrophage polarisation

**Fig. 2:**
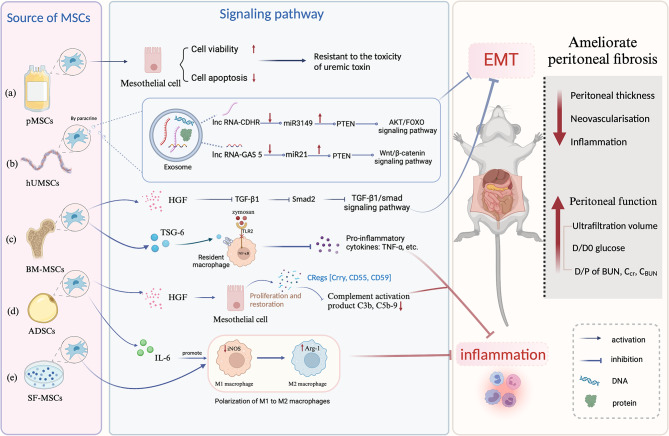
The possible mechanism of MSCs (Mesenchymal stem cells) to ameliorate peritoneal fibrosis. MSCs could reduce peritoneal fibrosis by two main mechanisms, including inhibiting EMT and inflammation. a: pMSCs could improve viability and reduce apoptosis in mesothelial cells, which can be more resistant to the toxicity of uremic toxin in rat models. b: hUMSCs could secrete exosomes through the paracrine pathway, and the gene lnc-CDHR and lnc-GAS5 in the exosomes can competitively bind to mi3149 to regulate PTEN through the AKT/FOXO signaling pathway and Wnt/β-catenin signaling pathway to inhibit EMT, thus reducing peritoneal fibrosis. c: BM-MSCs could secrete HGF through the TGF-β/Smad pathway to inhibit EMT and reduce peritoneal fibrosis. Simultaneously, BM-MSCs have the potential to release TSG-6, which interacts with local macrophage CD44 receptors to suppress the TLR2/NF-kB signaling pathway. This would decrease the release of pro-inflammatory cytokines and improve peritoneal fibrosis. d: ADSCs could secrete HGF to promote the proliferation and restoration of mesothelial cells, which could express abundant membrane complement regulators (Crry, CD55), further decreasing the complement activation products C3b, and C5b-9 to inhibit inflammation. ADSCs can also secrete IL-6, promoting the polarisation of M1 macrophages to M2 macrophages to inhibit inflammation and reduce peritoneal fibrosis. e: MSCs cultured in the serum-free medium can promote M2 macrophage polarisation to suppress inflammation. Created by BioRender.com

Macrophage polarization is the activation of macrophages in response to stimulation by pathogenic microbes, inflammatory reactions, cytokines, or certain physicochemical conditions, resulting in differentiation into various phenotypes [32]. Macrophage polarization is involved in the disease progression process, and when specific signals activate macrophages, they differentiate into distinct phenotypes that exert regulatory effects through a multitude of signaling pathways [33]. There are two main types of macrophage polarization: classically activated M1 and alternatively activated M2 [34]. M1 macrophages are known for their pro-inflammatory properties and secrete cytokines like IL-1β, iNOS, and TNF-α. On the other hand, M2 macrophages have anti-inflammatory properties and produce cytokines like IL-10, TGF-β, and Arg-1 [35]. Both M1 and M2 macrophages are critical in the pathogenesis of fibrosis. Therefore, macrophage polarization plays an important part in the progression of fibrotic diseases [36–40]. The researchers carried out cellular and animal experiments to test the hypothesis that promoting macrophage polarisation could alleviate PF. According to the study from Nagasaki et al. [41], SF-MSCs significantly reduced inflammation by stimulating the transition of proinflammatory M1 macrophages into immunosuppressive M2 macrophages. Yang et al. [7] constructed MGO-induced PF rat and cultured cells in vitro and found that TGF-β1 can stimulate ADSCs to secrete more IL-6 and increase the expression of the macrophage gene Arg-1, thereby promoting the polarization of M2 macrophages to decrease PF in rats. Research has indicated that inhibiting inflammation by inducing macrophage polarisation may help to alleviate PF, providing a new direction for the treatment of PF, but more supportive evidence is needed in this area, which requires further exploration of the involved mechanisms (Fig. 2).

### Secrete cytokines and growth factors

#### Secrete TSG-6, inhibit NF-κB signaling pathway

TSG-6 is a 30 kD glycoprotein with anti-inflammatory effects in various animal models [42, 43]. Studies have shown that the absence of the gene in transgenic mice increases inflammatory reactions, while overexpression reduces inflammation reactions [44, 45]. The protein’s anti-inflammatory activities are attributed to several effects, including its ability to bind to pro-inflammatory hyaluronan fragments, inhibiting the inflammatory cascade of proteases, and preventing the neutrophil inflow into inflammatory areas [46]. Nagasaki et al. [41] found that the STK2 serum-free medium enhanced the proliferation of MSCs and the secretion of TSG-6 in MSCs to suppress infiltration of inflammatory cells to suppress PF.

NF-κB is a key transcriptional regulator of the inflammatory response [47–49]. NF-κB can be activated by various stimuli that regulate inflammatory and immune responses and cell survival. The most powerful activators of NF-κB are inflammatory cytokines like tumor necrosis factor (TNF) or interleukin-1 (IL-1), as well as pathogen-derived molecules that trigger Toll-like receptors (TLRs) such as lipopolysaccharide (LPS), viral, and bacterial DNA and RNA [47, 50]. NF-κB is crucial for developing liver fibrosis [51–53]. Liao et al. [54] developed inflammatory and fibrotic models and discovered that isoliquiritigenin protects the kidney by inhibiting the Mincle/Syk/NF-κB signaling pathway. Choi et al. [6] constructed zymosan-induced mouse peritonitis to demonstrate that MSCs activated by inflammatory signals secrete the anti-inflammatory protein TSG-6 through TLR2/NF-κB signaling to attenuate zymosan-induced mouse peritonitis in resident macrophages. It has been discovered that TSG-6 interacts with macrophages through the CD 44 receptor to inhibit zymosan/TLR2-mediated nuclear translocation of the NF-κB, lowering pro-inflammatory factor secretion by macrophages and mitigating the inflammatory cascade that is released by resident macrophages and heightened by mesothelial cells or other potential peritoneal cells. In conclusion, secretion of TSG-6 by MSCs may play an important role in alleviating PF, and TSG-6 may act by inhibiting the infiltration of inflammatory cells through the NF-κB signaling pathway. And the NF-κB signaling pathway may provide a new direction for investigating the mechanism of PF (Fig. 2).

#### Secrete hepatocyte growth factor (HGF), inhibit TGF-β/Smad signaling pathway

HGF is a potent antifibrotic cytokine that blocks tubular epithelial-to-mesenchymal transition (EMT) induced by TGF-β [55]. HGF can also prevent bleomycin-induced pulmonary fibrosis in mice and inhibit the TGF-β/smad signaling pathway in lung cells [56]. TGF-β1 induces EMT in peritoneal mesothelial cells and contributes to the advancement of several kinds of fibrosis, including liver, lung, heart, and kidney [2, 57–59]. Ueno et al. [60]. have found through in vitro and animal experiments that MSCs can inhibit TGF-β1 production by secreting HGF through the paracrine pathway and improve TGF-β1-stimulated EMT in HPMCs through the TGF-β1/smad2 signaling pathway, thereby alleviating PF and reducing peritoneal functional impairment. Therefore, HGF may play an important role in alleviating PF, and the TGF-β signaling pathway may provide a new direction for MSC treatment of PF (Fig. 2).

#### Secrete HGF, inhibit complement deposition

Disorders of the complement activation system may be a contributing factor to peritoneal injury [61–63]. Kim et al. [64] constructed the Zy/scraping peritonitis model to investigate the relationship between complement activation and initial inflammation. They found that complement activation enhanced peritoneal inflammation in this peritoneal injury model. Rat ASCs were injected into the Zy/scraping peritonitis model, and it was found that HGF secreted by rat ASCs through the paracrine pathway may contribute to the repair of peritoneal mesothelial cell injury as well as peritoneal mesothelial cell proliferation and that the peritoneal mesothelial cells expressed abundant CReg (Crry, CD55, CD59), which could potentially prevent complement activation and the deposition of complement activation products like C3b and C5b-9 (Fig. 1).

### Secrete exosomes

Exosomes are tiny vesicles secreted by MSCs via the paracrine pathway that contain a variety of biomolecules, such as proteins, non-coding RNA, DNA, and mRNA, and act as important carriers in cell communication [28]. According to recent research, exosomes can alleviate fibrosis in the liver, lung, and kidney [29–31]. lnc RNA is widely found in fibrotic tissues such as the heart, liver, kidney, and lungs and plays an important role in the fibrotic process [65]. lnc RNA is present in the exosomes of MSCs [66]. Jiao et al. [67] discovered that hUMSCs’ exosomal lnc-CDHR binds competitively to miR-3149, regulating the target PTEN genes via AKT/FOXO signaling pathway to reduce EMT in HMrSV5. Huang et al. [68] suggested that exosomal lncRNA GAS5 competitively binds to miR21 and regulates PTEN via the Wnt/β-catenin pathway to inhibit EMT. Thus, Exosomes can alleviate PF, which might be attributed to the function of lncRNA. As an important carrier, lncRNA can play a vital role in alleviating PF. Due to the limited study, it is necessary for us to further explore the function of lncRNA in PF (Fig. 2).

## In vitro experiment

MSCs can reduce peritoneal mesothelial cell death, increase their activity and migration capacity, and promote their proliferation and injury repair, thereby inhibiting peritoneal mesothelial cell EMT and alleviating peritoneal injury (Table 1).

### MSCs can reduce mesothelial cell death and increase cell activity in vitro

MSCs can reduce the death of peritoneal mesothelial cells. Fan et al. [69] cocultured hUMSCs with PD-induced cell death in HPMCs and found that the hUMSCs could prevent morphological disturbances and apoptosis-like cell debris in HPMCs, improve cell viability in HPMCs and reduce the percentage of HPMCs death, which indicates a protective role of hUMSCs in peritoneal dialysis solution-induced HPMCs death. Du et al. [70] found that the secretome from pMSCs can significantly reduce peritoneal mesothelial cell death when exposed to PDS. Zhou et al. [71] incubated pMSCs-CM with H_2_O_2_-induced apoptosis and PDS-induced cell apoptosis in HPMCs and found that pMSC-CM could prevent cell death of cultured HPMCs (Table 1).

### MSCs can improve the migration capacity of mesothelial cells and promote injury repair in vitro

MSCs can enhance mesothelial cell migration. Wang et al. [72] constructed a mechanical injury model in vitro and discovered that MSCs could increase the migratory capacity and the proliferation of RPMCs in the early phase of injury. By co-culturing rat ASCs supernatant and peritoneal mesothelial cells, Kim et al. [64] found that the supernatant of rat ASCs could promote the proliferation of rat peritoneal mesothelial cells as well as the repair of injuries (Table 1).

### MSCs and their secretions inhibit EMT in mesothelial cells in vitro

MSCs inhibit EMT in peritoneal mesothelial cells. Guo et al. [73] co-cultured hUMSCs and SIRT1-modified hUMSCs with TGF-β1-stimulated Met-5 A cells respectively, and found that SIRT1-hUMSCs markedly inhibit EMT of Met-5 A cells compared to the hUMSCs. Specifically speaking, SIRT1-hUMSCs can significantly decrease the expression of mesenchymal and fibrotic markers such as Fibronectin, α-SMA, and Snail. Meanwhile, SIRT1-modified hUMSCs can restore the downregulate of expression of E-cadherin during EMT. Huang et al. [68] found that the conditional medium of hUMSCs could inhibit the EMT of HPMCs. Jiao et al. [67] discovered that hUMSCs exosomal lnc-CDHR binds to miR-3149 competitively and regulates the target PTEN genes’ repression to lessen EMT in HMrSV5 (Table 1).

## In Vivo experiment (animal experiment)

Studies have shown that long-term exposure of the peritoneal membrane to standard PD fluid with high glucose concentration results in morphological changes such as increased numbers of inflammatory cells, neovascularization, and submesothelial thickening, leading to UFF. MSCs, as a potential treatment, can reverse this change and protect peritoneal function [74] (Table 2).

**Table 2:** Summary of animal trials of mesenchymal stem cells for the treatment of peritoneal fibrosis

Author, year	Type of animal	Animal model	Stem cell type	Groups	Handling methods	Treatment effect	Outcome
Choi et al. (2011) [6]	Mice, C57BL/6J	Zymosan-induced mouse peritonitis	Human-BM-MSCs	1. HBSS 2. hMSCs 3. scr-siRNA hMSC 4. TSG-6-siRNA- hMSCs 5. 10ug TSG-6 6. 30ug TSG-6	1.6 × 10^6^ MSCs via IP. inject.	hUMSCs and 30ug rhTSG-6 treated: ↓: total number of cells and PMNs, the number of monocytes/macrophages	hMSCs and TSG-6 can reduce zymosan- induced peritonitis. There is no significant effect on TSG-6- siRNA-hUMCs treated.
Wang et al. (2012) [72]	SD-Rat	Acute peritoneal adhesion rat models	Rat-BM- MSCs	1. Serum-free medium 2. MSCs treated 3. MSC-CM	5 × 10^6^ BM-MSCs via tail vein inject.	BM-MSCs treated: ↓: adhesion formation, infiltration of MPO, macrophage cells (ED-1), FSP-1,TGF-β1 ↑: E-cadherin	Acute peritoneal adhesions were reduced by intravenously administered MSCs.
Wang et al. (2012) [86]	SD-Rat	Acute peritoneal adhesion rat models	Rat-BM- MSCs	1. Control 2. MSCs injected (IV) 3. MSCs injected (IP)	5 × 10^6^ MSCs via IP. inject. or IV. inject.	1. MSCs injected (IV): ↑: TSG-6 2. MSCs injected (IP): →: TSG-6	MSCs given intraperitoneally did not reduce peritoneal adhesion, while those injected intravenously significantly improved adhesion by secreting TSG-6.
				1. Medium injected 2. MSCs injected (IV) 3. siRNA-NC-MSCs injected (IV) 4. TSG-6-siRNA- MSCs injected (IV) 5. rmTSG-6-injected (IV)	Transfect TSG-6-siRNA into MSCs, TSG-6-siRNA-MSCs, TSG-6-siRNA-NC-MSCs or 3ng/ml rmTSG-6 were injected via the tail vein.	1. TSG-6-siRNA-MSC injected: →: TSG-6, Adhesion score, Peritoneal fibrosis score →: Fibrosis score 2. MSCs injected or rm TSG-6 injected: ↓: Adhesion score, Fibrosis score, Peritoneal fibrosis score	
Ueno et al. (2013) [60]	Fisher 344-Rat	CG-induced peritoneal fibrosis model	Rat-BM-MSCs	1. Control 2. CG + vehicle 3. CG + MSCs	1 × 10^7^ BM-MSCs via IP. inject.	MSCs treated: ↓: CD68 + cells, TGF-β1, α-SMA, peritoneal cell density and thickening, FSP-1, collagens I and III, pSmad2, D/P of BUN ↑: D/D0 of glucose	MSCs inhibit infiltration of inflammatory cells and TGF-β1/Smad pathway to reduce EMT to attenuate peritoneal fibrosis.
Baştuğ et al. (2013) [81]	Wistar albino rat	HG induced UFF in a chronic rat model of PD	Rat-BM-MSCs	1. Control 2. PUF-C 3. MSC 4. Placebo	1.5 × 10^6^ BM-MSCs via IP. inject.	↓: glucose mass transfer level, D/P of Cr, TGF-β, D/P_Na_, Submesothelial thickness, Fibrosis, inflammation, Neovascularization ↑: D/D0 glucose rate, UF capacity	MSCs can improve peritoneal function.
Wakabayashi et al. (2014) [76]	SD-Rat	CG-induced peritoneal fibrosis model	Rat-ASCs	1. CG 2. CG + ASCs	3 × 10^7^ ASCs via IP. inject.	ASC treated: ↓: TNF-α, IL-1β, MCP-1, Snail, α-SMA, SMC, Collagen III, HIF-1α, vascular density ↑: VEGF, PDGF-BB	ASCs transplantation significantly facilitate the peritoneal repair.
KIM et al. (2014) [64]	SD-Rat	Zy/scraping peritonitis	Rat-ASCs	1. rat ASCs (+) 2. rat ASCs (-)	6 × 10^6^ ASCs via IP. inject.	Rat-ASCs treated: ↑: anti-cytokeratin, CRegs (Crry, CD55, CD59) ↓: C3b, C5b-9, peritoneal thickness, infiltrative cells, neutrophils, counts of ED-1-possive cells	Rat ASCs can inhibit the accumulation of complement activation products to improve peritoneal damage.
FAN et al. (2016) [69])	SD-Rat	PD/MGO 3 W group	hUMSCs	1. Saline 3w 2. PD 3w 3. PD + MGO1w 4. PD + MGO2w 5. PD + MGO 3 W 6. PD + MGO 3 W + hUMSCs	1 × 10^7^ hUMSCs via IP. inject.	hUMSCs treated: ↓: ɑ-SMA, TGF-β, ED-1, D4/P4cr, inflammation, Collagen I, Neoangiogenesis, Relative glucose use in abdominal cavity ↑: D4/D0 glucose, drain volume	HUMSCs can significantly reduce the production of abdominal cocoons, peritoneal fibrosis, inflammation, neoangiogenesis, and UFF in rat models.
Li et al. (2018) [75]	Wistar rat	MGO-induced peritoneal fibrosis model	hUMSCs	1. Control 2. MGO 3. MGO + hUMSCs	2 × 10^6^ hUMSCs via tail vein inject.	MGO + hUMSCs treated: ↓: TGF-β1, Snail peritoneal thickeness, collagen type I/III, α-SMA ↑: D/D0 of glucose, E-cadherin	hUMSCs can improve the peritoneal function and attenuate the EMT.
Zhou et al. (2019) [71]	Wistar albino rat	PD induced chronic peritoneal dialysis rat model	pMSCs	1. Control 2. PD 3. PD + pMSC	1.2–1.5 × 10^6^ pMSCs via IP inject.	pMSCs treated: ↓: submesothelial thickness, blood vessels ↑: UF	Treatment with pMSC decreased blood vessel damage and PM damage as well as prevented UF loss.
Costalongaet al. (2020) [78]	Wistar rat	PF model developed in uremic rats	Rat ASCs	1. Control 2. CKD 3. PF 4. CKD + PF 5. CKD + PF + ASC	1 × 10^6^ ASCs via tail vein inject.	↓: inflammation peritoneal thickness, TGF-β, fibronectin, collagen III, α-SMA, IL-1β, TNF-α, IL-6	ASCs have anti-fibrotic effect and anti-inflammatory effect.
Guo et al. (2020) [73]	Wistar rat	MGO-PD-induced rat model	hUMSCs; SIRT1-hUMSCs	1. Control 2. PD 3. PD + hUMSCs 4. PD + hUMSCs- SIRT1	1 × 10^7^ hUMSCs via tail vein inject.	hUMSCs and hUMSCs-SIRT1 treated: ↑: D/D0 of glucose, SIRT1, Calretinin, ultrafiltration volume, ↓: IL-1β, MCP-1, peritoneal thickness, D/P of Cr, α-SMA, IL-6, TGF-β, pSmad3, TNF-α, Fibronectin, collagen III, Snail	SIRT1-hUMSCs can significantly improve the peritoneal function compare to hUMSCs.
Yang et al. (2021) [7]	SD-Rat	MGO-induced peritoneal fibrosis model	ADSCs; BM-MSCs	1. Control 2. MGO 3. MGO + BM-MSC 4. MGO + ADSC	1.5 × 10^6^ BM-MSCs or ADSCs via IP inject.	ADSCs or BM-MSCs treated: ↓: peritoneal thickness N-cadherin, α-SMA ↑: cytokeratin18, ARG-1/iNOS	ADSCs can significantly reduce the peritoneal thickness. ADSCs can significantly increase cytokeratin18 and ARG-1/iNOS.
Nagasaki et al. (2021) [41]	SD-Rat	CG-induced peritoneal fibrosis Model	SF-MSCs; 10%MSCs	1. Control 2. CG + Vehicle 3. CG + 10%MSCs 4. CG + SF-MSCs	5 × 10^6^ MSCs via IP inject.	SF-MSCs or 10%MSCs treated: ↓: cell desity, peritoneal thickness, α-SMA, TGF-β1, Collagen I and III, CD3^+^cells, CD68^+^cells ↑: CD163/CD68	Compared to 10%MSCs, SF-MSCs can significantly lessen the functional impairments of the peritoneal membrane.
Du et al. (2021) [70]	5/6 N × SD-Rat	PDS-induced PD uremia rat model	pMSCs	1. PBS 2. PDS 3. PDS + pMSCs 4. PDS + UC-MSCs	2 × 10^6^ pMSCs via IP inject.	pMSCs treated: ↓: submesothelial thickness, Scr, BUN, dilated tubules, number of blood vessels and capillarios, Urinary Pcr ↑: UF, Ccr, C_**BUN**_ D/D0 of glucose, →: reduction of Neoangiogenesis	pMSCs were superior to UC-MSCs in protecting the PM from structural change caused by PDS, but no significant change in neoangiogenesis.
			UC-MSCs	1. PBS 2. PDS 3. PDS + pMSCs 4. PDS + UC-MSCs	2 × 10^6^ pMSCs via IP inject.	UC-MSCs treated: →number of blood vessels and capillarios →UF, Ccr, C_BUN_ D/D0 of glucose →reduction of Neoangiogenesis	

Abbreviation; ↓: down-regulation; ↑: up-regulation; →: no significant change; IP.inject: intraperitoneal injection; IV inject: intravenous injection; MSCs: mesenchymal stem cells; hMSCs: human bone marrow mesenchymal stem cells; hUMSCs: human umbilical cord blood mesenchymal stem cells; ASCs: adipose-derived mesenchymal stem cells; BM-MSCs: bone marrow mesenchymal stem cells; SD-Rat: Sprague-Dawley rats; TSG-6: TNF-α–stimulated gene 6 protein; TGF-β1: transforming growth factor-β1; PMN: Polymorphonuclear cells; CM: conditional medium; MPO: Myeloperoxidase; FSP-1: fibroblast-specific protein-1; D/P of BUN: dialysate-to-plasma ratio of blood ureanitrogen; D/D0 of glucose: the peritoneal absorption of glucose from the dialysate; CG: chlorhexidine gluconate; EMT: epithelial-to-mesenchymal transition; HG: High concentration of glucose; UFF: ultrafiltration failure; PD: peritoneal dialysis; PMNs: Poly Morphonuclear Neutrophil; α-SMA: α-smooth muscle actin; MCP-1: monocyte chemoattractant protein-1; SMC: submesothelial compact; HIF-1α: hypoxia inducible factor-1α; PDGF-BB: platelet-derived growth factor-BB; MGO: methylglyoxal; D4/P4 creatine, the dialysate to plasma creatinine ratio at 4 h after administration; D4/D0 glucose: the ratio of the glucose levels in D0 and D4 in drained dialysate; D/P of cr: the ratio of dialysate and plasma creatinine concentration; PM: peritoneal membrane; pMSCs: PD effluent-derived mesenchymal stromal cells; ARG-1: Arginine-1; iNOS: inducible nitric oxide synthase; Scr: serum creatinine; Ccr: clearance of Creatine; C_BUN_: clearance of blood urea nitrogen; VEGF: vascular endothelial-derived growth factor; IL-1β: interleukin-1β; IL-6: interleukin 6; BUN: blood urea nitrogen; SF: serum-free medium

### MSCs can inhibit EMT in animal models

MSCs significantly suppressed the accumulation of myofibroblasts and macrophages, as well as the expression of mesenchymal markers (α-SMA and FSP-1) and extracellular matrix (ECM) proteins (collagens I and III), leading to amelioration of PF [60]. Guo et al. [73] and Li et al. [75] constructed an MGO-PD-induced rat model and investigated that hUMSCs alleviate EMT in the peritoneal injury rat model. Wakabayashi et al. [76] developed a CG-induced PF model and discovered that ASCs could inhibit the EMT to reduce experimental PF (Table 2).

### MSCs have anti-fibrotic and anti-inflammatory properties in animal models

Several studies have demonstrated that MSCs have antifibrotic and anti-inflammatory effects. Yang et al. [7] and Nagasaki et al. [41] induced a PF model and found that ADSCs and BM-MSCs both can suppress PF. Yu et al. [77] constructed peritoneal injury mouse models induced by 2.5%PDF + LPS and found that BM-MSC-Exos alleviates PF related to PD and relieves peritoneal inflammation and angiogenesis in mice. Costalonga et al. [78] constructed PF models developed in uremic rats and found that ASCs have antifibrotic and anti-inflammatory effects. Specifically speaking, ASC therapy dramatically decreased macrophage and T-cell infiltration and improved the development of PF in the PF rat model. ASC infusion prevented PF by lowering the number of peritoneal myofibroblasts and changing the expression of genes involved in ECM synthesis (Table 2).

### MSCs can ameliorate peritoneal function in animal models

Several studies have demonstrated that MSCs can improve peritoneal function in animal models by inhibiting peritoneal thickness, improving ultrafiltration [75, 79–81], and the absorption rate of glucose from the dialysate (D/D0 of glucose) [41, 77, 80]. Ueno et al. [60] discovered that MSCs can improve the D/D0 of glucose and the transport rate of blood urea nitrogen from the plasma (D/P of BUN) to inhibit functional impairment. Guo et al. [73] discovered that SIRT1-hUMSCs markedly improved ultrafiltration volume, D/P of Cr, and D/D0 of glucose in PD-treated rats.

## Clinical application of MSCs

Due to the scarcity of clinical trials on the use of stem cells to treat PF, the following are three studies in which stem cell therapy was utilized to improve peritoneal function in PD patients. hUMSCs have the advantages of strong proliferation and differentiation ability, easy access to materials, and low immunogenicity, and have better application prospects than other stem cells [82]. Jiang et al. [83] found that hUMSC treatment partially improved clinical indicators of continuous ambulatory peritoneal dialysis (CAPD) patients. Within three months after hUMSCs transplantation, experimental results showed a significant increase in hemoglobin, erythropoietin, and albumin levels, decreased C-reactive protein levels, and marked improvement in cystatin C and urine volume (Table 3).

Adipose tissue is a prospective source for autologous cell-based treatment since it is more accessible than bone marrow [84]. A study conducted by Ahmadi et al. [8] suggests that injecting Autologous ADSCs may lead to a slight improvement in UF capacity and mild enhancement in peritoneal membrane function. The study found a slight increase in both systemic and peritoneal levels of CA125 and a minor decrease in gene expression levels of TGF-β, α-SMA, and FSP-1. Alatab et al. [85] conducted a study on PD patients suffering from UFF who were on CAPD for at least two years and discovered that systemic delivery of ADSCs to PD patients was feasible and well tolerated, with no severe adverse events or catheter-related problems noted. Therefore, from the above results, it is clear that applying MSCs in clinical trials can improve peritoneal function indices to a certain extent and is safe and reliable. Due to insufficient clinical trials, the exploration of MSCs is inadequate. More clinical trials are needed in the future, including the following aspects: identifying the most suitable types of MSCs, exploring how to maximize the effect of MSCs, and improving the convenience and efficiency of MSC preparation technology (Table 3).

## Discussion

Prolonged PD can lead to the development of PF, which can force patients to withdraw from PD [3]. It is crucial to slow down the process of PF. MSCs have been studied in clinical trials and basic experiments due to their ability to self-regenerate, modulate the immune system, and repair tissue damage [14]. MSCs can repair tissue damage, but their mechanism is controversial. Initially, it was thought that MSCs repair tissues by engrafting and differentiating to replace injured cells [19–23]. Nowadays, MSCs have been proven to exert their beneficial effects by secreting cytokines and growth factors through the paracrine pathway or cell-to-cell contacts. Exosomes, which are important secretions produced by MSCs, have been found to significantly reduce fibrosis in different tissues such as the liver, lung, kidney, and peritoneum [29–31, 67, 68]. However, a recent study found that exosomes derived from PD effluents could result in peritoneal damage by transmitting molecules such as proteins [86]. Therefore, it is important to identify the specific components of exosomes that are beneficial or harmful and to understand their role in regulating peritoneal function.

Different sources of MSCs may have varying effects on improving peritoneal function. Yang et al. [7] argued that ADSC has a more significant antifibrotic effect than BM-MSC in reducing peritoneal membrane thickness because the researchers found that ADSC can release more IL-6 than BM-MSC and IL-6 as an important component during the process of M2 macrophage polarization, which can significantly reduce the PF. Du et al. [70] found that pMSCs were more effective than hUMSCs in protecting the peritoneal membrane and remnant kidneys in 5/6Nx rats, which was mainly explained by the fact that pMSCs exhibited greater resistance to the toxicity of uremic toxins present in uremic rats and were more protective of peritoneal mesothelial cells from death. The effect of different injection modalities of MSCs on relieving peritoneal adhesions may be different. Wang et al. [87] investigated that MSCs given intraperitoneally did not reduce peritoneal adhesion, while those injected intravenously significantly improved adhesion. The reasons may be that intravenously injected MSCs, which accumulated mainly in the lung, can survive for 7 days, are rarely phagocytosed by macrophages, and secrete TSG-6 within 12 h. In contrast, intraperitoneally injected MSCs, accumulating mainly in the spleen and can survive for only 4 h, are subsequently phagocytosed by macrophages and do not secrete TSG-6, thus failing to exert a therapeutic effect.

The therapeutic effects of MSCs in rat models and clinical trials cannot be fully equated. This may be related to the immune compatibility between the donor and recipient, MSC dosing, and the fitness of culture-adapted MSCs [88]. In animal models, MSCs are usually administered intravenously at 50 million MSC/kg/dose, but in most clinical trials, MSCs are usually administered intravenously at 1–2 million cells/kg/dose and no more than 12 million cells/kg. Therefore, there is a disproportionate relationship between dose and body weight. Thus, the disproportionate relationship between dose and body weight suggests a possible reason for the discrepancy between the efficacy of animal studies and clinical trials [89]. In addition, there may be some challenges in using MSCs in clinical trials: (1) different sources of MSCs have different extraction methods, and there are no standardized guidelines on this; (2) there are some technical difficulties in culturing and expanding MSCs; (3) different injection modality and different dosage may cause different effects, so exploring the most appropriate injection modality and dosage is important for the future; (4) The transportation and storage conditions of MSCs can impact the therapeutic effectiveness of MSCs in clinical trials, and it is necessary to conduct further study in the future. Although there are many unresolved problems in using MSCs, current clinical studies have shown that MSCs are safe and feasible, with no serious adverse effects and no catheter-related complications reported [85, 90, 91].

Since the effects of MSCs in animal experiments are not the same as in actual clinical trials, it is necessary to use technical skills to improve the therapeutic effects of MSCs. Combining stem cell therapy and biotechnology is one of the potential fields for tissue damage and repair. Huang et al. [92] found that SIRT1 significantly alleviates renal fibrosis in rat models of chronic kidney disease and murine mesangial cells. SIRT1 knockdown increases renal fibrosis and destroys renal function, whereas SIRT1 overexpression decreases TGF-β-induced extracellular matrix production and expression [92]. Guo et al. [73] constructed MGO-PD-induced rat models and found that SIRT1-modified hUMSCs can markedly reduce the PF in rat models. In addition, Huerta et al. [93] found that E-selectin gene-modified MSCs could accelerate wound healing compared to MSC and phosphate-buffered saline treatment group. Studies have indicated that a hypoxic environment is beneficial to stem cell survival rate [94]. Trisnadi et al. [95] found that hypoxic conditions-induced MSCs might significantly reduce the TGF-β level in peritoneal adhesion rat models compared to the normal MSCs. MSCs cultured in serum medium may contribute to inflection and increase the risk of transmitting viral disease [96, 97]. So, the serum-free medium is important to clinical application [98, 99]. Nagasaki et al. [41] used the serum-free conditional medium to culture MSCs, and the results indicate that SF-MSC was more effective in inhibiting PF than using 10% MSC (10% serum conditional medium). Furthermore, serum-free MSC culture has many benefits, including shortened culture cycles, reduced risk of infection from serum components, no longer having to check for variations in serum batches, and enhanced cell proliferation stability and efficiency. This provides valuable insight for the in vitro expansion of MSCs in clinical settings [96, 97].

This review provides an overview of all studies in vitro and in vivo on MSCs in treating dialysis-induced PF. Due to the lack of in-depth research in this field, there is a paucity of research study on the mechanism. Therefore, a large number of studies in vitro and in vivo are needed to demonstrate the efficacy of MSCs. Furthermore, Tables 1, 2 and 3 summarises the different sources of MSCs, different treatments of MSCs, and different animal models of fibrosis induction, MSC doses, and different indicators of peritoneal function, as well as the signaling pathways involved in MSCs. These can provide data support for subsequent researchers to get a quick overview of the current state of research, save time in collecting literature, and conduct further advance study.

## Conclusion

This review summarises the latest research progress on the alleviation of PF by MSCs, including the mechanism of MSCs in alleviating PF, cellular experiments, animal experiments, and the clinical application of MSCs. Numerous studies have demonstrated that MSCs alleviate PF mainly through the paracrine pathway. The following signaling pathways were found to be involved in the alleviation of PF by MSCs: TGF-β/smad signaling pathway, AKT/FOXO signaling pathway, Wnt/β-catenin signaling pathway, TLR/NF-κB signaling pathway. MSCs can alleviate PF by secreting exosomes, which contain genes that regulate miRNA action targeting PTEN to inhibit EMT and alleviate PF. MSCs can also alleviate PF by inhibiting the infiltration of inflammatory cells, inducing macrophage polarisation, stimulating TSG-6 secretion by MSCs, and secreting HGF to inhibit complement deposition. In addition, serum-free cultured MSCs may help to suppress inflammation. The effects produced by different sources of MSCs are different, and the effects of different injection modalities in relieving peritoneal adhesions may also vary. Therefore, the field of MSCs to alleviate PF is currently understudied, and more mechanisms still need to be explored. MSCs provide a new direction for progress in the treatment of PF in dialysis patients.

## Data Availability

Data sharing is not applicable to this article, as no datasets were generated or analyzed during the current study.

## Abbreviations

ADSCs: Adipose-derived mesenchymal stem cells
Alb: Albumin
Arg-1: Arginine-1
BM-MSCs: Bone marrow mesenchymal stem cells
BUN: Blood urea nitrogen
BMP-7: Bone morphogenic protein 7
BMI: Body mass index
CA125: Cancer antigen 125
Ccr: Creatinine clearance rate
CM: Conditional medium
CG: Chlorhexidine gluconate
C_BUN_: Clearance of blood urea nitrogen
D/P of BUN: Dialysate-to-plasma ratio of blood urea nitrogen
D/Pcr: The ratio of dialysate and plasma creatinine concentration
EMT: Epithelial-to-mesenchymal transition
ECM: Extracellular matrix
EPO: Erythropoietin
Exo: Exosome
FSP-1: Fibroblast-specific protein-1
FWT: Free water transport
GLU D/P: The glucose dialysate-to-plasma ratio
Hb: Hemoglobin
HG: High concentration of glucose
HIF-1α: Hypoxia-inducible factor-1α
HPMCs: Human peritoneal mesothelial cells
Hs-CRP: High-sensitivity C-reactive protein
HGF: Hepatocyte growth factor
HMrSV5: Human peritoneal mesothelial cell line
hUMSCs: Human umbilical cord-derived mesenchymal stem cells
iNOS: Inducible nitric oxide synthase
IP.inject: Intraperitoneal injection
IV.inject: Intravenous injection
IL-1β: Interleukin-1β
IL-6: Interleukin 6
LPS: Lipopolysaccharides
MSCs: Mesenchymal stem cells
10%MSCs: MSCs cultured in a medium containing 10% fetal bovine serum
MPO: Myeloperoxidase
MCP-1: Monocyte chemoattractant protein-1
MGO: Methylglyoxal
Met-5A: An immortalized human pleural mesothelial line
NC: Negative control
OCG: Osmotic conductance to glucose
PMN: Polymorphonuclear cells
PD: Peritoneal dialysis
PMNs: PolyMorphonuclear neutrophil
PF: Peritoneal fibrosis
PDGF: Platelet-derived growth factor
PM: Peritoneal membrane
pMSCs: PD effluent-derived mesenchymal stromal cells
PDS: PD solution
RPMC: Rat peritoneal mesothelial cells
Rat-MC-CM: Rat mesothelial-CM
SD-Rat: Sprague-Dawley rat
α-SMA: α-Smooth muscle actin
SMC: Submesothelial compact
Scr: Serum creatinine
SF: Serum-free medium
SF-MSCs: MSCs in serum-free medium
TSG-6: TNF-α–stimulated gene 6 protein
TGF-β1: Transforming growth factor-β1
THP-1 cells: A human monocytic cell line
UFF: Ultrafiltration failure
UFSP: Ultrafiltration- small pore
UF: Ultrafiltration
UFT: Ultrafiltration total
VEGF: Vascular endothelial-derived growth factor
ZO-1: Zonula occludens-1
